# Correction: Associations of gamma-glutamyl transferase with cardio-metabolic diseases in people living with HIV infection in South Africa

**DOI:** 10.1371/journal.pone.0259483

**Published:** 2021-10-28

**Authors:** Kim A. Nguyen, Nasheeta Peer, Andre P. Kengne

The following information is missing from the Funding statement: Furthermore the project is part of the EDCTP2 programme supported by the European Union (grant number TMA2017GSF-1962—CaDERAL). The correct Funding statement is as follows: The work reported herein was made possible through funding by the South African Medical Research Council through its Division of Research Capacity Development under the SAMRC Intramural Postdoctoral Programme from funding received from the South African National Treasury. Furthermore the project is part of the EDCTP2 programme supported by the European Union (grant number TMA2017GSF-1962—CaDERAL). The content hereof is the sole responsibility of the authors and do not necessarily represent the official views of the SAMRC or the funders.
