# Supplementary material for: Associations of gamma-glutamyl transferase with cardio-metabolic diseases in people living with HIV infection in South Africa
Source: PLoS One. 2021 Feb 5;16(2):e0246131. doi: 10.1371/journal.pone.0246131 (PMC7864415; doi:10.1371/journal.pone.0246131)
Supplement: Attachment — Submitted filename: Response to Reviewers_PONE-D-20-26437_19.12.2020.docx [file pone.0246131.s001.docx]

**PONE-D-20-26437**

**Associations of gamma-glutamyl transferase with cardio-metabolic diseases in people living with HIV infection in South Africa**

**PLOS ONE**

Dear Dr Ethan Morgan

We are grateful to the reviewers for providing constructive feedback, which has improved our manuscript. Together with this letter we submit a revised version in the track and change format for further consideration for publication in the journal. Details of the revisions are shown in the Response to Reviewers Table below:

| **Editorial comments** |  | **Responses** |
| --- | --- | --- |
|  | 1. Please ensure that your manuscript meets PLOS ONE's style requirements, including those for file naming. The PLOS ONE style templates can be found at  [https://journals.plos.org/plosone/s/file?id=wjVg/PLOSOne_formatting_sample_main_body.pdf](https://protect-za.mimecast.com/s/cBJ5CAnXJoT9kJlpf9yYyO) and  [https://journals.plos.org/plosone/s/file?id=ba62/PLOSOne_formatting_sample_title_authors_affiliations.pdf](https://protect-za.mimecast.com/s/UbVFCBgXKpIVYJRWijp0il) | Thank you for the note. |
|  | 2. Please provide further details on sample size and power calculations. | We have added this information in the revised manuscript (Material and Methods section, Data analysis, lines 142-145.)  For a z-value of 1.96 (corresponding to a 95% confidence interval), and a sample size of 709 participants, our study had a margin of error of 0.07% to detect a prevalence of hypertension of 1%. This indicated our study was well-power as the accepted margin is of 5%. |
|  | 3. In statistical methods, please clarify whether you corrected for multiple comparisons. | Please see the revised manuscript, lines 136-139 |
|  | 4. In your Data Availability statement, you have not specified where the minimal data set underlying the results described in your manuscript can be found. PLOS defines a study's minimal data set as the underlying data used to reach the conclusions drawn in the manuscript and any additional data required to replicate the reported study findings in their entirety. All PLOS journals require that the minimal data set be made fully available. For more information about our data policy, please see [http://journals.plos.org/plosone/s/data-availability](https://protect-za.mimecast.com/s/43yaCDRZMrcB4rJYtlhdi6). | The data will be uploaded to the SAMRC MEDAT Data Repository and the link provided once the paper has been accepted for publication. We trust that this is acceptable. |
|  | Upon re-submitting your revised manuscript, please upload your study’s minimal underlying data set as either Supporting Information files or to a stable, public repository and include the relevant URLs, DOIs, or accession numbers within your revised cover letter. For a list of acceptable repositories, please see [http://journals.plos.org/plosone/s/data-availability#loc-recommended-repositories](https://protect-za.mimecast.com/s/4YxCCElXNvuWEo04UQbZwZ). Any potentially identifying patient information must be fully anonymized.  We will update your Data Availability statement to reflect the information you provide in your cover letter. | Please see our response above. |
| **Reviewer #1: COMMENTS** |  |  |
| Main comments | 1. The authors highlight that the association between GGT and metabolic syndrome/ CVD risk has already been established in general population. What would be the reason to expect such an association would not apply to PLWH? | PLWH may differ metabolically from general populations, as has been shown in their development of cardio-metabolic diseases, which has been attributed not only to traditional risk factors but also to HIV-related factors. That places them at an increased risk for cardio-metabolic diseases. Unlike general population, CVD and metabolic diseases in PLWH are likely due to the combined effects of HIV infection, extended exposure to ART and traditional cardiovascular risk factors. In addition, PLWH often developed cardio-metabolic disease risk at younger ages. Therefore, the association between GGT and metabolic syndrome/ CVD risk, established in general population, is not generalisable to PLWHIV. We have included the following text on lines 55-57 in the paper:  “Considering that the development of CVDs in the HIV-infected are attributable to both traditional risk factors and HIV-related variables, findings in general populations may not be generalisable to the HIV-infected.” |
|  | 2. I am sorry to say that I am not sure what would be the clinical use of the information of this meticulous analysis. Are we proposing that we should be screening patients for CVD/ DM/ etc when the GGT is raised? Or, should we dismiss further management of raised GGT when any of the identified conditions are present? | The literature shows that there is a need to improve the identification of individuals at high risk for CVD/cardio-metabolic diseases, including in PLWHIV. If a wide range of studies confirm the association between GGT and CVD/cardio-metabolic diseases, including in PLWHIV, it would suggest the potential use of GGT as a cardiovascular biomarker. Note that this is independent of its utility in liver disease.  Please see lines 311-312, the revised manuscript. |
|  | 3. In practice, most of the patients with raised GGT have elevated ALT and AST too (indeed Table 1 also agrees with this statement). We interpret liver profile and not just one of the liver enzyme to define clinical abnormality. How do we propose to use the study information for management of those patients? | In the absence of a probable/likely diagnosis of liver disease in a patient with elevated liver enzymes, our study adds to the body of literature, specifically in PLWHIV, to suggest an alternate diagnosis. Note that current evidence shows that elevated GGT in the normal range is a significant predictor of incident hypertension or diabetes (Lee et al, 2003; Ford et al, 2008). Furthermore, some studies found only GGT, but not ALT and/or AST, was associated with CVD risk (Fraser et al, 2009).  Please see lines 67-69, the revised manuscript.  Ford ES, Schulze MB, Bergmann MM, Thamer C, Joost H-G, Boeing H. Liver Enzymes and Incident Diabetes. Diabetes Care. 2008 Jun 1;31(6):1138–1143.  Lee D-H, Jacobs DRJ, Gross M, Kiefe CI, Roseman J, Lewis CE, et al. Gamma-glutamyltransferase is a predictor of incident diabetes and hypertension: the Coronary Artery Risk Development in Young Adults (CARDIA) Study. Clin Chem. 2003 Aug;49(8):1358–1366.  Fraser A, Harris R, Sattar N, Ebrahim S, Davey Smith G, Lawlor DA. Alanine aminotransferase, gamma-glutamyltransferase, and incident diabetes: the British Women’s Heart and Health Study and meta-analysis. Diabetes Care. 2009 Apr;32(4):741–750. |
|  | 4. I am not sure the quarters of GGT used for the analysis are clinically relevant. The study's GGT quarters Q1, Q2 and Q3 are all within reference range and normal. Should we not carry out the analysis between all within reference range compared with those with elevated range according to the MACS classification of abnormal liver enzymes? | We thank the reviewer for this suggestion. However, as mentioned above, elevated GGT in the normal range is a significant predictor of incident hypertension or diabetes. Therefore, analyses by GGT quartiles provide more insight when evaluating the associations of CVD/cardiometabolic disease with GGT. We trust this is acceptable. |
|  | 5. I notice the authors have acknowledged that they did not investigate the roles of viral hepatitis and cART medications. I think these are quite important omissions particularly considering the background prevalence of the infections in South Africa. | We agree with the reviewer, and this is a limitation of our paper, which we have included under our limitations. Unfortunately, we do not have data on viral hepatitis status and ARV drugs and are unable to evaluate these variables. We have accounted for the duration of HIV infection, which may be correlated with the duration of ART. These are demonstrated on lines 297-302 in the revised manuscript. |
|  | 6. Unless I am mistaken, the analysis included even one unit of alcohol per time of study definition in the alcohol consumption group. This is strictly correct however not clinically helpful. Essentially what we need is to identify the threshold of consumption beyond which GGT would rise. | We note the reviewer’s comment. However, we only determined alcohol as a binary yes/no variable for current alcohol intake and adjusted for this in our analyses. |
|  | 7. I notice that the majority of the study patients were women? Any reason for this? Would this have caused a bias? | Studies in South Africa invariably tend to recruit more women compared with men for various reasons and this has been described in many studies. More women tend to use health care services and are also more willing to participate in research than men in South Africa. We have included this as a limitation of our study.  Please see lines 295-297, the revised manuscript. |
|  | 8. As stated earlier, I am not a statistician. I am interested to understand how nearly every factor investigated in this study had significant association with raised GGT? Some of the findings don't make immediate sense to me; e.g. why would CD4 count have such a relationship with raised GGT? | The significant associations of cardio-metabolic diseases with GGT in this study mostly reflect those that have been demonstrated in general populations. Similarly, HIV-specific factors have been shown to be associated with cardio-metabolic diseases and may also be involved in the pathway between GGT and cardio-metabolic diseases. However, this exploration is beyond the scope of the current study. |
| **Minor comments** | 1. Use of Framingham score in a population of non-White patients mostly living in rural areas may create a major issue with the validity of the calculated CVD risk. | Unfortunately, in the absence of South African or ethnic specific equations for black populations, the default is to use the Framingham risk score. This has been included as a limitation in our study (lines 304-306 in the revised manuscript). |
|  | 2. The results sections are full of mathematical evidence with little narrative on their clinical implications. | We have amended the results section and hope it is now easier to follow. The narrative on the clinical implications is presented in the Discussion. |
| **Reviewer #2: COMMENTS** |  |  |
|  | 1. The authors mentioned at the beginning of the Discussion that the study was the first ever to investigate the association of GGT with cardiovascular risk factors in HIV infected Africans (Lines 228-229 and 291-293). This is not correct; the EndoAfrica study in the Western Cape Province of South Africa has done a similar study already. (1. [https://bmcinfectdis.biomedcentral.com/articles/10.1186/s12879-020-05173-6](https://protect-za.mimecast.com/s/FCbRCKO7WBFqVxM7S2-URP)) (2. [https://bmcinfectdis.biomedcentral.com/articles/10.1186/s12879-016-2158-y](https://protect-za.mimecast.com/s/p4NZCLg1XDIP6MwBS5st4j)) I will suggest that the authors take a look at the studies to see how to re-frame their study/story. | We have amended the Discussion section as follows: “To our knowledge, this study is among the first studies to investigate the associations of serum GGT with CVD risk factors in HIV-infected Africans. A recently published paper described higher GGT levels in HIV-infected vs. HIV-uninfected participants but did not examine the associations of GGT with cardio-metabolic diseases (Strijdom et al. 2017; Fourie et al. 2020).” We trust this is acceptable**.** Please see lines 221-224, the revised manuscript.  Strijdom H, De Boever P, Walzl G, Essop MF, Nawrot TS, Webster I, et al. Cardiovascular risk and endothelial function in people living with HIV/AIDS: design of the multi-site, longitudinal EndoAfrica study in the Western Cape Province of South Africa. BMC Infect Dis [Internet]. 2017;17(1):41.  Fourie CMT, Botha-Le Roux S, Smith W, Schutte AE, Breet Y, Mels CMC, et al. Vascular function and cardiovascular risk in a HIV infected and HIV free cohort of African ancestry: baseline profile, rationale and methods of the longitudinal EndoAfrica-NWU study. BMC Infect Dis [Internet]. 2020;20(1):473. |
|  | 2. The authors also observed that the association of GGT with almost all the parameters that were investigated was similar to what has been shown in various populations; both disease conditions and control groups. This suggests that the study is not adding anything new to subject. | We have included the following text, which we hope adds clarity: “Nevertheless, the evidence from the literature on the associations of serum GGT with cardio-metabolic diseases in general populations cannot be generalised to HIV-infected populations. The associations in the HIV-infected may differ because of the different pathways involved in the development of cardio-metabolic diseases in the HIV-infected, i.e., traditional risk factors together with HIV-specific factors, which could differentially influence the relation of GGT and cardio-metabolic outcomes.’ (Lines 234-239, the revised manuscript).  Hsue PY, Deeks SG, Hunt PW. Immunologic basis of cardiovascular disease in HIV-infected adults. J Infect Dis [Internet]. 2012 Jun;205 Suppl 3(Suppl 3):S375–82. Available from: https://pubmed.ncbi.nlm.nih.gov/22577211.  Grinspoon SK, Grunfeld C, Kotler DP, Currier JS, Lundgren JD, Dubé MP, et al. State of the science conference: Initiative to decrease cardiovascular risk and increase quality of care for patients living with HIV/AIDS: executive summary. Vol. 118, Circulation. 2008. p. 198–210.  Friis-Møller N, Reiss P, Sabin CA, Weber R, Monforte A d’Arminio, El-Sadr W, et al. Class of antiretroviral drugs and the risk of myocardial infarction. N Engl J Med. 2007 Apr;356(17):1723–35. |
| **Abstract:** | 3. Referring to HIV infected patients/subjects as “a sample” is not correct and should be corrected throughout the manuscript (E.g. Lines 18 and 36-37 in the Abstract section). | The corrections were done throughout the manuscript. |
| **Material and Methods:** | 4. Lines 93-94 in the Method section. The sentence does not indicate the number of times the BP was taken, which is important in this case. | Thank you. We have included ‘thrice’ to indicate that the blood pressure was taken 3 times. (line 97 in the revised manuscript) |
|  | 5. Lines 99-102. The sentence is not clear and needs to be revised. | Thank you. We rephrased the sentence for more clarity. Please see the revised manuscript, Measurements session, lines 103-105. |
|  | 6. Lines 102-104. The sentence needs to be referenced. | The reference has been included, thank you (line 108). |
|  | 7. Line 106. I will suggest that you either say "as previously described" or you would have to put the mathematical equation. | We changed the texts into mathematical equation. Please see line 111 in the revised manuscript. |
| **Results:** | 8. Lines 139-140. I will suggest that the first sentence in the Results section is moved to the Methods section. | We respectfully disagree with the reviewer. The sample realisation belongs in the Results and not the Methods section. |
| General: | 9. There are minor typographical errors that needs to be corrected. E.g. Line 105. The word “Chemiluminescence” has an extra “e”. | We thank the reviewer for his/her comment and have corrected the spelling accordingly. Line 109 in the revised manuscript. |
|  |  |  |
| **Notes from Journal:** | *While revising your submission, please upload your figure files to the Preflight Analysis and Conversion Engine (PACE) digital diagnostic tool,* [*https://pacev2.apexcovantage.com/*](https://protect-za.mimecast.com/s/OOp6CO7X1kTAWrZ1tR3XmL)*. PACE helps ensure that figures meet PLOS requirements. To use PACE, you must first register as a user. Registration is free. Then, login and navigate to the UPLOAD tab, where you will find detailed instructions on how to use the tool. If you encounter any issues or have any questions when using PACE, please email PLOS at* [*figures@plos.org*](mailto:figures@plos.org)*. Please note that Supporting Information files do not need this step.* | Thank you for the notes. |
|  |  |  |

We hope that this submission meets your requirements.

Many thanks and kind regards

On behalf of the co-authors
