# Supplementary material for: Associations of gamma-glutamyl transferase with cardio-metabolic diseases in people living with HIV infection in South Africa
Source: PLoS One. 2021 Feb 5;16(2):e0246131. doi: 10.1371/journal.pone.0246131 (PMC7864415; doi:10.1371/journal.pone.0246131)
Supplement: Attachment — Submitted filename: Response to Reviewers_PONE-D-20-26437R1_7.1.2021.docx [file pone.0246131.s002.docx]

*PLOS ONE

Dear Dr Ethan Morgan*

*Many thanks to yourself and the reveiwer for reviewing our revised manuscript. We wish to submit a revised version for further consideration for publication in the journal. Details of the revisions are shown below.*

**Comments to the Author**

1. If the authors have adequately addressed your comments raised in a previous round of review and you feel that this manuscript is now acceptable for publication, you may indicate that here to bypass the “Comments to the Author” section, enter your conflict of interest statement in the “Confidential to Editor” section, and submit your "Accept" recommendation.

Reviewer #2: (No Response)

2. Is the manuscript technically sound, and do the data support the conclusions?

The manuscript must describe a technically sound piece of scientific research with data that supports the conclusions. Experiments must have been conducted rigorously, with appropriate controls, replication, and sample sizes. The conclusions must be drawn appropriately based on the data presented.

Reviewer #2: Partly

**Response:** Thank you.

3. Has the statistical analysis been performed appropriately and rigorously?

Reviewer #2: Yes

**Response**: Thank you.

4. Have the authors made all data underlying the findings in their manuscript fully available?

The [PLOS Data policy](https://protect-za.mimecast.com/s/MK8ICY6YkxFD30nri9pVcw) requires authors to make all data underlying the findings described in their manuscript fully available without restriction, with rare exception (please refer to the Data Availability Statement in the manuscript PDF file). The data should be provided as part of the manuscript or its supporting information, or deposited to a public repository. For example, in addition to summary statistics, the data points behind means, medians and variance measures should be available. If there are restrictions on publicly sharing data—e.g. participant privacy or use of data from a third party—those must be specified.

Reviewer #2: Yes

**Response**: Thank you.

5. Is the manuscript presented in an intelligible fashion and written in standard English?

PLOS ONE does not copyedit accepted manuscripts, so the language in submitted articles must be clear, correct, and unambiguous. Any typographical or grammatical errors should be corrected at revision, so please note any specific errors here.

Reviewer #2: Yes

**Response**: Thank you.

6. Review Comments to the Author

Please use the space provided to explain your answers to the questions above. You may also include additional comments for the author, including concerns about dual publication, research ethics, or publication ethics. (Please upload your review as an attachment if it exceeds 20,000 characters)

Reviewer #2: The authors have addressed most of the comments that were raised, however, the major concern has not been addressed.

Even though the authors have incorporated the suggested EndoAfrica project references, they still have it in the Introduction section that “the association between gamma-glutamyl transferase (GGT) and CVD risk, while confirmed in general populations, has not been investigated in the HIV-infected“. I think this needs to be corrected.

**Response:** We revised the sentence as “For example, the association between gamma-glutamyl transferase (GGT) and CVD risk, while confirmed in general populations, has been little studied in the HIV-infected.” Please see line 61 in the revised manuscript.

Secondly, I do not agree to the revisions made to the first sentence of the Discussion section which says that “this study is among the first to investigate the associations of serum GGT with CVD risk factors in HIV-infected Africans”. The study is not the first and the authors would just have to accept that fact.

**Response**: We replaced the sentence as “To our knowledge, there is an on-going cohort study by Strijdom et al. aiming to determine whether HIV-infection and ART are associated with cardiovascular risk variables and changes in vascular structure and function in adults living in South Africa.” Please read lines 221-223 in the revised manuscript.

7. PLOS authors have the option to publish the peer review history of their article ([what does this mean?](https://protect-za.mimecast.com/s/lrATCZ4Glys7MmkJSyshTl)). If published, this will include your full peer review and any attached files.


If you choose “no”, your identity will remain anonymous but your review may still be made public.


**Do you want your identity to be public for this peer review?** For information about this choice, including consent withdrawal, please see our [Privacy Policy](https://protect-za.mimecast.com/s/96n0C1jpmqF6pjPVhY4g0x).

Reviewer #2: No

[NOTE: If reviewer comments were submitted as an attachment file, they will be attached to this email and accessible via the submission site. Please log into your account, locate the manuscript record, and check for the action link "View Attachments". If this link does not appear, there are no attachment files.]

While revising your submission, please upload your figure files to the Preflight Analysis and Conversion Engine (PACE) digital diagnostic tool, [https://pacev2.apexcovantage.com/](https://protect-za.mimecast.com/s/G-P1C2RqnrcVkxBMSMyav9). PACE helps ensure that figures meet PLOS requirements. To use PACE, you must first register as a user. Registration is free. Then, login and navigate to the UPLOAD tab, where you will find detailed instructions on how to use the tool. If you encounter any issues or have any questions when using PACE, please email PLOS at [figures@plos.org](mailto:figures@plos.org). Please note that Supporting Information files do not need this step.

*In compliance with data protection regulations, you may request that we remove your personal registration details at any time.* [*(Remove my information/details)*](https://protect-za.mimecast.com/s/hjFQC3lrovuXm8ynCYnuYG)*. Please contact the publication office if you have any questions.*

*Kind regards,*

*On behalf of all authors*
